# Novel CYP11B-ligand [^123/131^I]IMAZA as promising theranostic tool for adrenocortical tumors: comprehensive preclinical characterization and first clinical experience

**DOI:** 10.1007/s00259-021-05477-y

**Published:** 2021-07-03

**Authors:** Britta Heinze, Andreas Schirbel, Lukas Nannen, David Michelmann, Philipp E. Hartrampf, Christina Bluemel, Magdalena Schneider, Ken Herrmann, Heribert Haenscheid, Martin Fassnacht, Andreas K. Buck, Stefanie Hahner

**Affiliations:** 1 Division of Endocrinology and Diabetes, Department of Medicine I, University Hospital of Wuerzburg, University of Wuerzburg, Oberduerrbacher Strasse 6, 97080 Wuerzburg, Germany; 2 Department of Nuclear Medicine, University Hospital of Wuerzburg, University of Wuerzburg, Oberduerrbacher Strasse 6, D-97080 Wuerzburg, Germany; 3 Department of Nuclear Medicine, Medical Faculty, University Hospital Essen, Hufelandstrasse 55, D-45122 Essen, Germany; 4 Comprehensive Cancer Center Mainfranken, University of Wuerzburg, Josef-Schneider-Str. 2, D-97080 Wuerzburg, Germany

**Keywords:** CYP11B enzymes, Adrenocortical carcinoma, Adrenal incidentaloma

## Abstract

**Purpose:**

Adrenal tumors represent a diagnostic and therapeutic challenge. Promising results have been obtained through targeting the cytochrome P450 enzymes CYP11B1 and CYP11B2 for molecular imaging, and [^123/131^I]iodometomidate ([^123/131^I]IMTO) has even been successfully introduced as a theranostic agent. As this radiopharmaceutical shows rapid metabolic inactivation, we aimed at developing new improved tracers.

**Methods:**

Several IMTO derivatives were newly designed by replacing the unstable methyl ester by different carboxylic esters or amides. The inhibition of aldosterone and cortisol synthesis was tested in different adrenocortical cell lines. The corresponding radiolabeled compounds were assessed regarding their stability, in vitro cell uptake, in vivo biodistribution in mice, and their binding specificity to cryosections of human adrenocortical and non-adrenocortical tissue. Furthermore, a first investigation was performed in patients with known metastatic adrenal cancer using both [^123^I]IMTO and the most promising compound (R)-1-[1-(4-[^123^I]iodophenyl)ethyl]-1H-imidazole-5-carboxylic acid azetidinylamide ([^123^I]IMAZA) for scintigraphy. Subsequently, a first endoradiotherapy with [^131^I]IMAZA in one of these patients was performed.

**Results:**

We identified three analogues to IMTO with high-affinity binding to the target enzymes and comparable or higher metabolic stability and very high and specific accumulation in adrenocortical cells in vitro and in vivo. Labeled IMAZA exhibited superior pharmacokinetic and imaging properties compared to IMTO in mice and 3 patients, too. An endoradiotherapy with [^131^I]IMAZA induced a 21-month progression-free interval in a patient with rapidly progressing ACC prior this therapy.

**Conclusion:**

We developed the new radiopharmaceutical [^123/131^I]IMAZA with superior properties compared to the reference compound IMTO and promising first experiences in humans.

**Supplementary Information:**

The online version contains supplementary material available at 10.1007/s00259-021-05477-y.

## Introduction

**Fig. 1:**
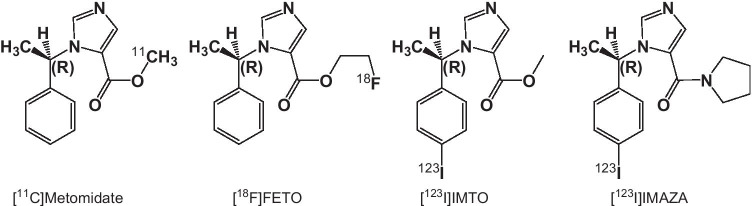
Chemical structures of [^11^C]metomidate, [^18^F]FETO, [^123^I]IMTO, and [^123^I]IMAZA

Adrenal tumors belong to the most frequent human neoplasias with an age-dependent prevalence of 2–10% [1–3]. The spectrum of potential differential diagnoses largely varies; it includes non-functioning adrenocortical adenomas, representing the most frequent tumor entity, but also malignant adrenal primary tumors or metastases from other malignancies [2]. While non-functioning adrenocortical adenomas do not require further treatment, malignant or hormone-producing tumors are usually removed by surgery. Adrenocortical carcinoma (ACC) is a malignancy with an overall poor prognosis and non-satisfactory treatment in advanced disease [4–9]. The only approved drug in advanced ACC is mitotane, being associated with significant adverse effects and a response rate of maximum 24% [10, 11]. Early diagnosis and surgical resection are the only curative option for these patients. However, a significant number of adrenal incidentalomas cannot be completely characterized by conventional imaging methods like computed tomography (CT) or magnetic resonance imaging (MRI) [12]. Functional imaging providing more information on the molecular background of a tumor may be of help to fill in this diagnostic gap. The radiotracers [^11^C]metomidate (MTO), [^18^F]fluoroetomidate (FETO), and [^123^I]iodometomidate (IMTO) (chemical structures of these tracers and of the new compound [^123^I]IMAZA shown in Fig. 1) selectively bind to adrenocortical CYP11B enzymes and have been successfully used in humans for specific molecular imaging of adrenocortical tissue [13–23].

High uptake of [^123^I]IMTO was reported not only for primary tumors but also for metastatic lesions in ACC. In a recent series of 11 patients with advanced ACC, we demonstrated a therapeutic potential of treatment with [^131^I]IMTO, resulting in a lasting stabilization of the disease in the majority of patients, with only few adverse effects [24].

Although [^123^I]IMTO represents a suitable tool for adrenal imaging and therapy, studies of the metabolic stability revealed rapid degradation in patients by esterases. IMTO derivatives resistant to rapid degradation but still maintaining high avidity to adrenal CYP11B enzymes could allow lower radiation exposure during imaging and improved imaging quality due to lower background activity and higher effective doses in targeted treatment of ACC lesions. Therefore, we aimed at establishing a new theranostic tool targeting adrenocortical tumors by development of IMTO derivatives with improved properties regarding target binding, tracer stability, and target to background ratios.

## Materials and methods

### Chemistry and radiochemistry

For the preparation of the derivatives, IMTO (compound **1)** was saponificated to the corresponding acid **2** and converted into esters **3** and **4** and the carboxylic amide **5** with the help of coupling agents. The trimethylstannyl precursors were synthesized from the corresponding selected iodinated arenes. Radioiododestannylation was chosen for labeling of the stannylated precursors and delivered each tracer in high yields (> 90%). Experimental details of chemistry and radiochemistry of the most important compounds IMTO, IMAZA, **3**, and **4** are presented in the supplement.

### Evaluation of CYP11B1 and CYP11B2 inhibition in Y1-Cyp11B1 and Y1-Cyp11B2

The human adrenocortical cell line NCI-H295 and the murine adrenocortical cancer cell line Y1 transfected with either human CYP11B1 or CYP11B2 were used for determination of IC_50_ values as already described [17].

### Analysis of metabolic inactivation by liver microsomes in vitro

Metabolic degradation was determined by co-incubation of the tracers with human liver microsomes (Sigma, M0317). Experiments were conducted in triplicates. Incubation mixtures consisted of liver microsomes (0.2 mg/ml), the respective ^125^I-labeled compound (0.1 MBq), MgCl_2_ (3.3 mM), and NADPH (1.3 mM) in a total volume of 0.6 ml K_3_PO_4_ puffer (25 mM, pH 7.2). The reaction was started with the addition of NADPH and incubated at 37 °C. At predefined time points, aliquots were removed, and the reaction was terminated by adding 160 µL ice-cold methanol. After centrifugation (5000 × g, 4 °C for 4 min), the supernatant was analyzed by radio-HPLC (see experimental details and Supplementary Fig. 1 in Supplement).

### Cell uptake studies

NCI-H295 cells (*n* = 250,000 per vial) were incubated with 0.1 MBq of the respective ^125^I-labeled tracer at 37 °C, 5% CO_2_. For the blocking experiment, the cells were co-incubated with etomidate. Aliquots were taken and immediately placed into a 4 °C ice bath. After 5 min, the solution was centrifuged and rinsed twice with PBS/0.5% Tween 80, and the collected rinse solution was measured in a gamma counter. Assays were performed in triplicates. The intracellular distribution of the respective ^125^I-labeled inhibitors and the specific binding to mitochondrial membranes were verified using a commercially available kit (Qiagen Qproteome Mitochondria isolation).

### Autoradiographic binding experiments to human tissue cryosections

Human tissues were quick frozen in liquid nitrogen to block further biological processes including protein degradation and stored at –80 °C. Frozen samples from adrenocortical carcinoma (ACC; *n* = 3), aldosterone-producing adenoma (APA; *n* = 2), and cortisol-producing adenoma (CPA; *n* = 2), normal adrenal, kidney, and liver were sectioned into 20-µm-thick tissue slices, thaw-mounted onto Superfrost slides (PAP PEN, Zymed® Laboratories) and stored at –80 °C until processing. For the experimental set up, the tissue slices were pre-incubated for 5 min at room temperature with buffer solution (pH 7.4, 50 mM Tris). Afterwards, the slices were directly incubated with TRIS HCl buffer solution containing 2% BSA. After 1-h blocking, slides were treated with 0.1 MBq of the different compounds. As controls, slides were incubated with 0.1 MBq of the compounds in 10 µM of the non-radioactive compound to assess binding specificity. In the following, slides were washed twice with 50 mM Tris HCl buffer solution (pH 7.4). For exposure, samples were placed on Phosphor Imager plates for 30 min in dedicated lead-shielded cassettes. Autoradiographic images were analyzed with a CR35BIO Image plate scanner, and data analysis was performed with Amide software version 0.9.0.

### Animal experiments

Six- to 8-week old male CD-1 mice received 0.1 MBq [^125^I]IMTO [17] or the ^125^I-labeled IMTO-analogues as intravenous injection in the tail vein. At predefined time points, mice were sacrificed (min. *n* = 5 per time point). Blood and urine were collected, and several organs were excised and weighed, and tracer uptake was determined by measurement in a γ-counter.

### Diagnostic imaging with [^123^I]IMTO and [^123^I]IMAZA

Three patients (#1, #2, and #3) were investigated by [^123^I]IMAZA (administered doses, 117, 163, and 182 MBq, respectively) and subsequently by [^123^I]IMTO (176, 169, and 146 MBq, respectively). The time interval between the imaging studies was at least 5 days. Scintigraphic imaging was performed using a Siemens Symbia dual-head gamma camera equipped with medium-energy parallel-hole collimators [18, 19]. Activity concentration was measured in whole blood samples drawn at 2 min, 5 min, 15 min, 1 h, 2.5 h, 5 h, and 23 h after the administration. Plasma activity was analyzed in patient #1.

### Use of [^131^I]IMAZA for endoradiotherapy

Patient #1 had experienced disease progression with a more than fourfold increase in her dominant lesions within only 4 months after a phase of disease stabilization after standard treatment with mitotane and eight courses of combination chemotherapy with etoposide, doxorubicin, and cisplatin. She received therapeutic doses with 28.2 GBq [^131^I]IMAZA 1 month and with 30.5 GBq [^131^I]IMAZA 7 months after the evaluation with [^123^I]IMAZA. Prior to the first treatment, the absorbed dose to the blood was measured with a tracer activity of 72 MBq [^131^I]IMAZA as a surrogate for the exposure of the dose-limiting organ, the red bone marrow. The absorbed dose to the blood was estimated from assessments of the activity kinetics in total body and blood up to 48 h when the mean activity concentrations had dropped to 5% of the values at 5 min after the administration in the total body and to 1% in the blood. During therapy, the total body activity retention was measured with a survey meter in the ceiling over the patient’s bed. Gamma camera whole body scans accompanied by blood sampling were acquired starting 4 days post-administration after the first and 2 days after the second therapy until dismissal. SPECT/CT imaging was performed after 4 and 5 days, respectively.

Tumor evaluation was performed by [^18^F]FDG PET/CT at baseline and 2, 5, 8, 11, 14, and 21 months after administration of the first treatment dose. Subsequently, the patient declined further follow-up imaging or additional therapeutic interventions.

### Statistical analysis

IC_50_ values were calculated from all values using the program GraphPad Prism5®. All results are expressed as mean ± standard deviation.

## Results

### Effects of compounds on secretion of cortisol and aldosterone in vitro

The majority of the new compounds inhibited cortisol and aldosterone secretion in a dose-dependent manner both NCI-H295 cells and Y1 cells transfected with either human CYP11B1 or CYP11B2, indicating target enzyme binding. The IC_50_ values for inhibition of cortisol and aldosterone in Y1 cells are displayed in Table 1.

**Table 1:**
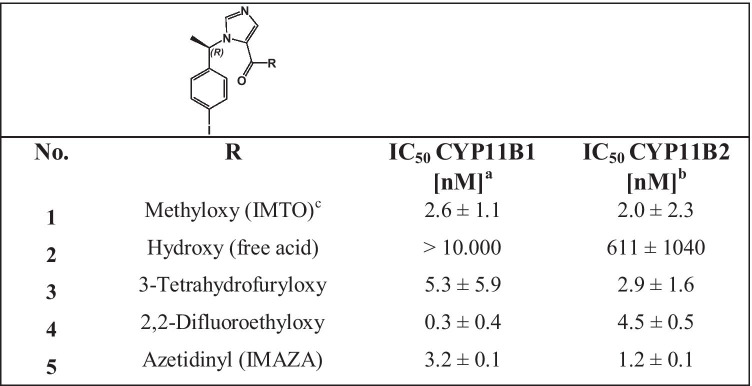
Inhibition of CYP11B1 and CYP11B2 by three carboxylic esters, the corresponding free acid, and one carboxylic amide. ^a,b^IC_50_ values for Cyp11B1 and Cyp11B2 activity in stably transfected Y1-hsCyp11B1^a^ and Y1-hsCyp11B2^b^ cells, substrate deoxycortisol, and 11-deoxycorticosterone (1 µM); ^c^reference compound. For all compounds, the mean value of at least three individual experiments, each performed in duplicates is displayed.

### Metabolic stability of radiolabeled compounds in vitro

The metabolic stability of [^125^I]IMTO and three new tracers was determined by radio-HPLC; results are listed in Table 2. The esters **3** and **4** exhibited nearly equal or even better metabolic stability than [^125^I]IMTO. The most stable compound was [^125^I]IMAZA showing a more than threefold longer half-life than [^125^I]IMTO.

**Table 2:** In vitro T_1/2_ in human liver microsomes for [^125^I]IMTO and three new compounds. The in vitro T_1/2_ value of the reference [^125^I]IMTO and [^125^I]IMAZA represents mean ± S.D. for triplicate determinations in three independent experiments. In vitro T_1/2_ values of the remaining compounds represent mean ± S.D. for triplicate determinations in one independent experiment

No	Compound	T_1/2_ [min]
1	Iodometomidate (IMTO)	7.3 ± 3.6
3	3-Tetrahydrofurylester	7.4 ± 2.8
4	2,2-Difluoroethylester	7.5 ± 0.87
5	Azetidinylamide (IMAZA)	26.1 ± 5.5

### Effects of compounds on steroidogenesis in NCI-H295 cells

**Fig. 2:**
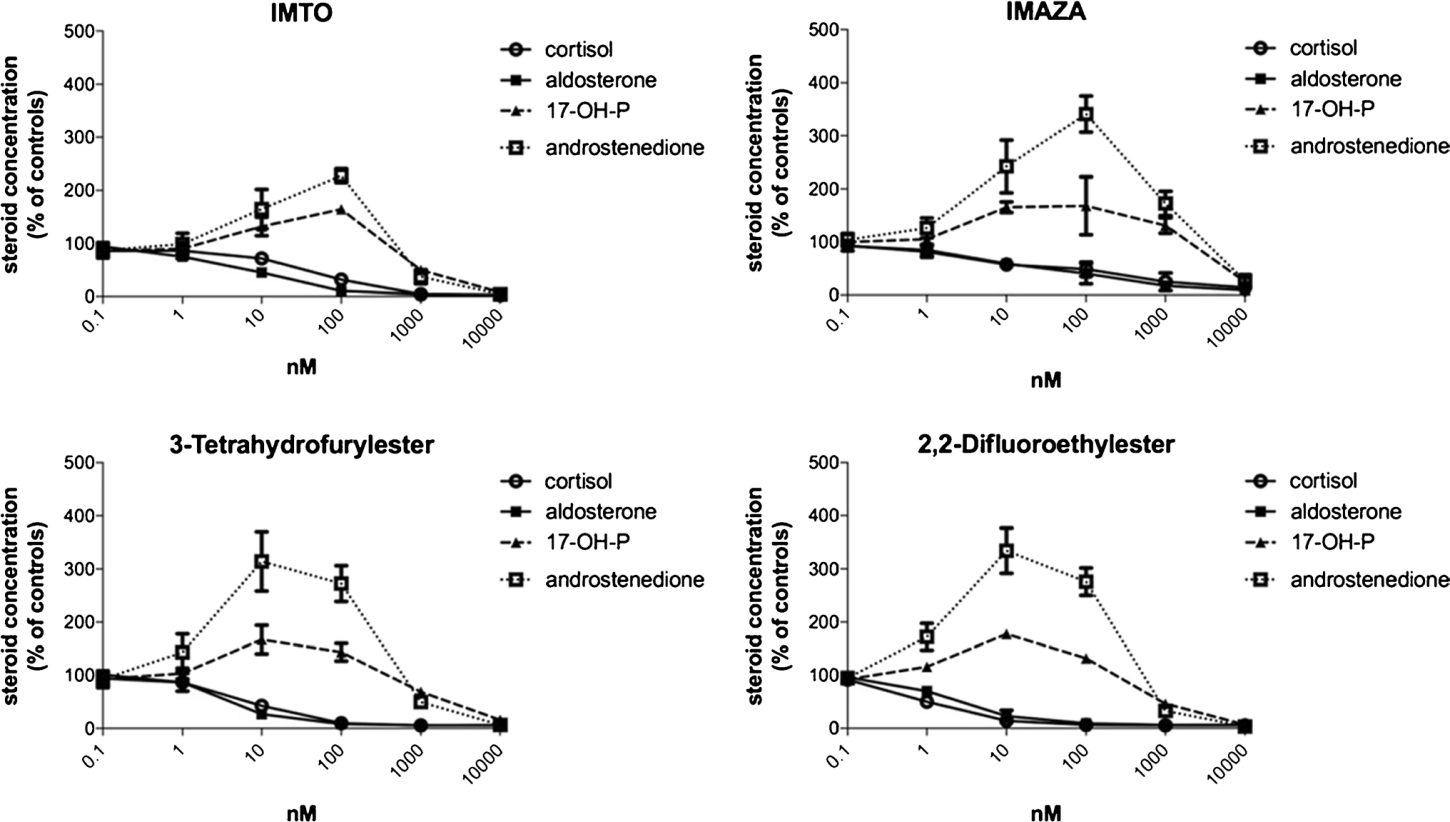
Steroid secretion in NCI-H295 cells after 48-h incubation with IMTO, IMAZA, 3-tetrahydrofurylester, and 2,2-difluoroethylester (0.1–10,000 nM). NCI-H295 cells were incubated for 48 h with the four compounds. Each experiment was performed in triplicates. At the end of the incubation period, steroid hormones (cortisol, aldosterone, 17-OH-P, and androstenedione) were determined in the cell supernatant by commercially available assays

The compounds 3-tetrahydrofurylester **3** and 2,2-difluoroethylester **4** and IMAZA exhibited both strong enzyme inhibition and at least comparable metabolic stability to IMTO, so they were additionally investigated in NCI H295 cells. An accumulation of the upstream steroids 17-OH-progesterone and androstenedione was observed at lower concentrations of the compounds (0.1–10 nM) due to the block of both CYP11B enzymes. At higher concentrations, the production of the investigated hormones was completely stopped, most likely due to additional inhibition of side chain cleavage enzyme (CYP11A1), similar to the known effect of metomidate (Fig. 2).

### Cellular uptake of selected radiolabeled compounds

**Fig. 3:**
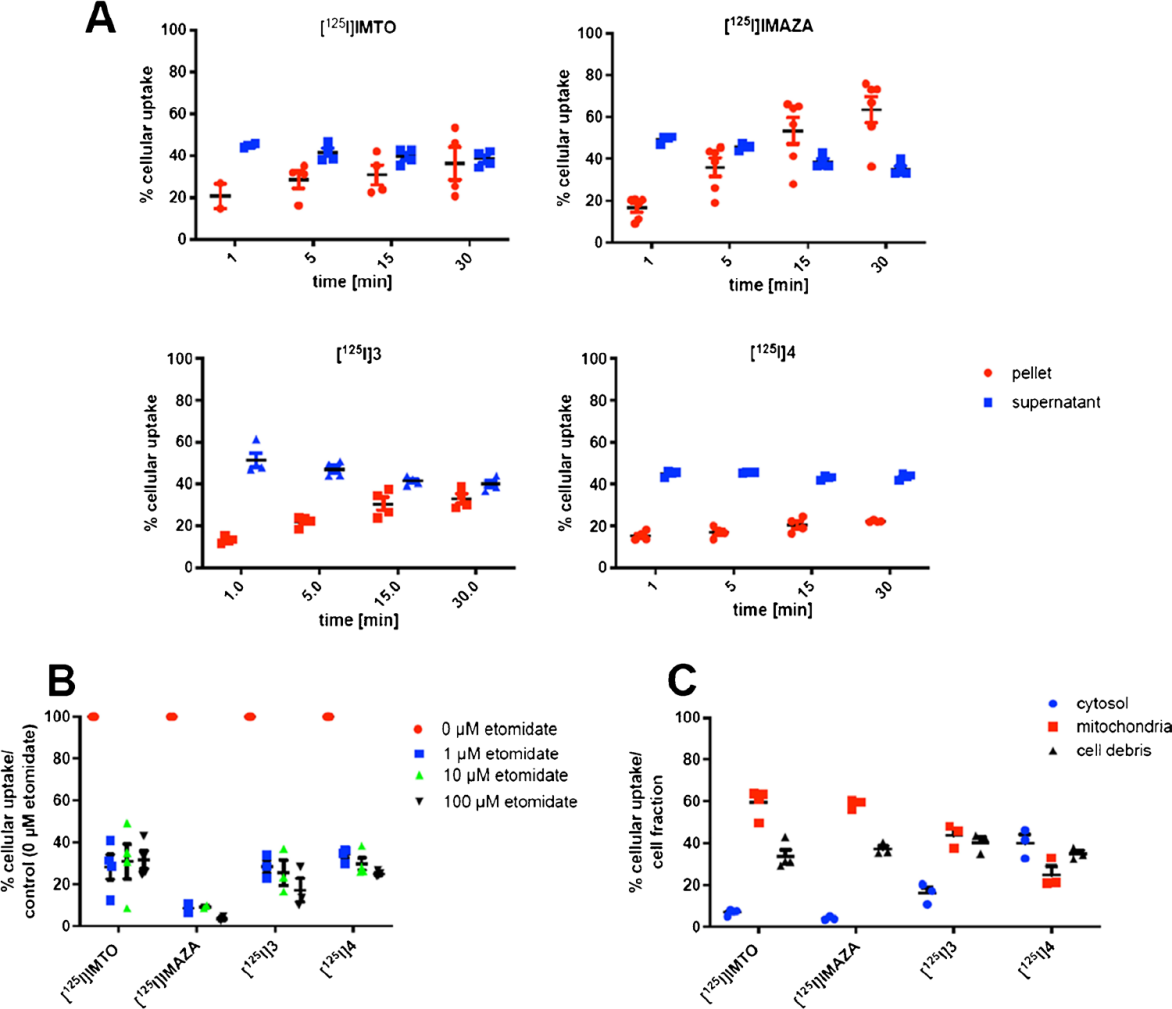
**a** In vitro uptake kinetics of [^125^I]IMTO, [^125^I]IMAZA, [^125^I]**3**, and [^125^I]**4** in NCI-H295 cells. **b** Cellular uptake of [^125^I]IMTO, [^125^I]IMAZA, [^125^I]**3**, and [^125^I]4 in NCI-H295 cells with and without co-incubation with etomidate. Cells were incubated with 0.1 MBq of the respective compounds and co-incubated with (0.1 µM, 10 µM, 100 µM) etomidate to monitor the specific uptake (*n* = 3). **c** Cellular subfractionation of NCIH295 cells exposed to 0.1 MBq [^125^I]IMTO, [^125^I]IMAZA, [^125^I]**3**, and [^125^I]4 for 30 min at 37 °C. Distribution of the tracer compounds among the respective cellular subfractions

The intracellular uptake in human adrenocortical NCI-H295 cells was analyzed for [^125^I]IMTO and the compounds exhibiting the highest metabolic stability: [^125^I]**3**, [^125^I]**4**, and [^125^I]IMAZA. [^125^I]IMTO **1** showed a time-dependent uptake up to 36.4% after 30 min. The two esters [^125^I]**3** and [^125^I]**4** demonstrated a slightly lower intracellular accumulation than [^125^I]IMTO at 30 min, and [^125^I]IMAZA exhibited the highest uptake in NCI-H295 cells (Fig. 3a).

To assess the specificity of all compounds, uptake experiments were furthermore performed after co-incubation with etomidate, a known inhibitor of CYP11B1 and CYP11B2. With the lowest concentration of 1 µM etomidate, the intracellular uptake for [^125^I]IMTO was 28.2%, for [^125^I]IMAZA 8.6%, for [^125^I]**3** 28.4%, and for [^125^I]**4** 34.3% (Fig. 3b). With the highest concentration of 100 µM etomidate, the uptake values showed only a small additional increase in blockage. For [^125^I]IMAZA, the uptake was almost completely blocked with the highest concentration of etomidate indicating specific binding (Fig. 3b).

### Tracer binding to cellular subfractions

As both target enzymes are localized at the inner mitochondrial membrane, cellular subfractionation experiments were performed using human NCI-H295 cells. As shown in Fig. 3c, [^125^I]IMTO demonstrated high accumulation in the mitochondrial subfraction (59.4%) and a lower accumulation in the nuclei, cell debris subfraction (33.6%), and in the cytosolic fraction (7%). Results were similar for [^125^I]IMAZA, while [^125^I]**3** and [^125^I]**4** showed a lower accumulation in the mitochondrial subfraction. Thus, particularly for [^125^I]IMTO and [^125^I]IMAZA, a high and specific binding to the CYP11B enzymes as targets localized in the mitochondrial cell fraction may be assumed (Fig. 3c). The purity of each fraction was subsequently confirmed by Western blot.

### Biodistribution in CD-1 mice

**Fig. 4:**
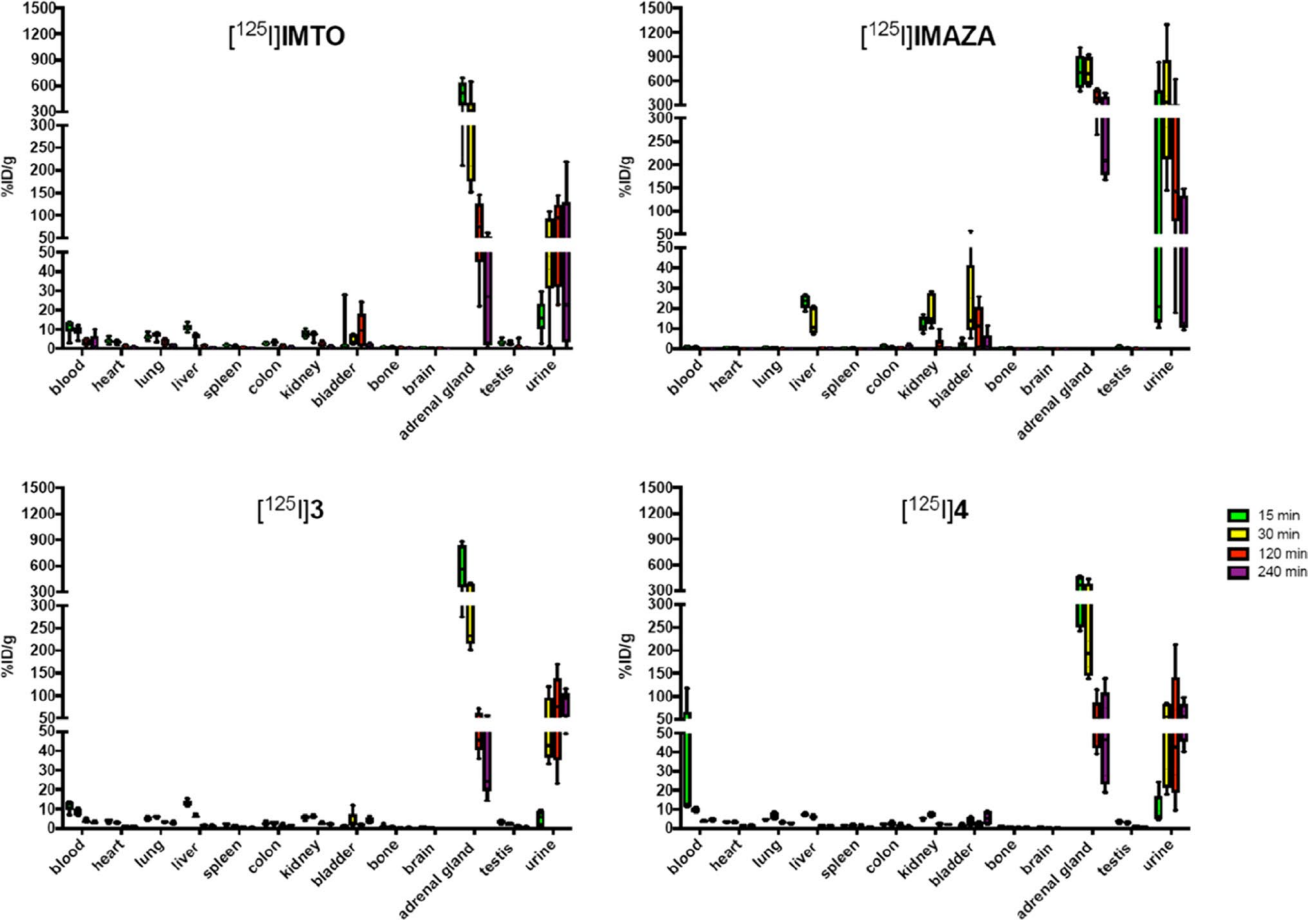
Biodistribution of [^125^I]IMTO and the new compounds [^125^I]IMAZA**,** [^125^I]**3**, and [^125^I]**4** in male CD-1 mice. Mice were injected i.v. with 0.1 MBq of respective compounds. Ex vivo organ dosimetry was performed at predefined time points 15 min, 30 min, 120 min, and 240 min after tracer injection. Results are expressed as percentage of injected dose per gram organ (%ID/g). Exact numbers on uptake in the adrenal as %ID/g for the compounds are given in the supplemental data

Biodistribution of [^125^I]IMTO, [^125^I]IMAZA, [^125^I]**3**, and [^125^I]**4** was investigated in male CD-1 mice. As previously described for [^125^I]IMTO, a fast and specific uptake in the adrenal tissue could be observed, which was followed by a rapid clearance within 240 min (Fig. 4). Also, for the other compounds, a specific uptake in the adrenal gland was observed. [^125^I]IMAZA not only showed an outstandingly high uptake in the adrenal gland, but also demonstrated extremely low uptake values in the non-target organs (Fig. 4 and Supplementary Table 1).

### Autoradiographic experiments

**Fig. 5:**
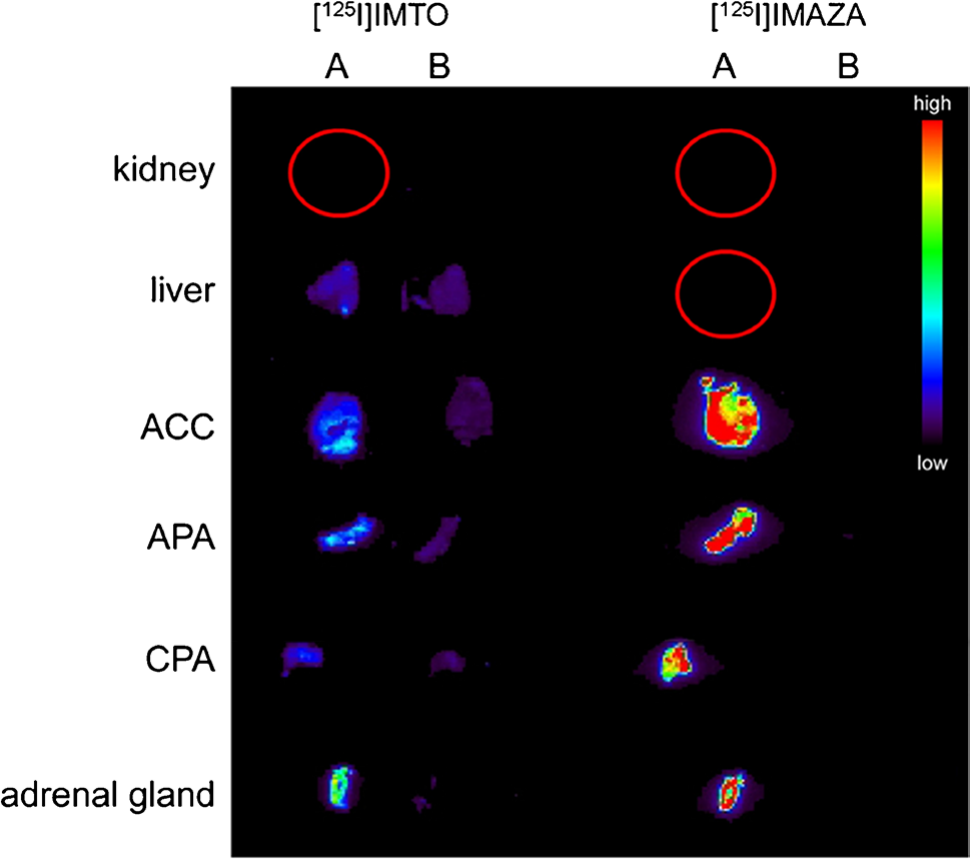
Ex vivo imaging of normal adrenal gland, adrenocortical tumors, and control tissue (kidney, liver). Autoradiographic visualization of [^125^I]IMTO and [^125^I]IMAZA accumulation in the normal adrenal gland, in adrenocortical tumors (ACC, APA, CPA), kidney, and liver as control tissue (sections with 20 µm slice thickness) (representative example). Red colors indicate highest uptake of radioligand; blue/dark blue colors indicate no uptake or full competition. The red circles show the position of the organs that are not shown in the imaging. A = 0.1 MBq tracer, B = 0.1 MBq tracer plus non-radioactive 10 µM inhibitor

Based on the results from in vitro screening, [^125^I]IMAZA was found to be the most promising compound and was further tested in binding experiments to human tissue sections. Autoradiographic visualization of [^125^I]IMAZA showed very strong and specific binding to ACC, APA, and CPA tissue, but no binding was detected for the control tissue (liver, *n* = 2; kidney, *n* = 1; Fig. 5). The signal was distinctively stronger for [^125^I]IMAZA than for the reference compound [^125^I]IMTO, indicating improved binding to human adrenocortical tissue (Fig. 5).

### [^123^I]IMTO and [^123^I]IMAZA in three patients with advanced ACC

After the administration of [^123^I]IMTO or [^123^I]IMAZA, renal clearance was fast for both compounds but more rapid for [^123^I]IMAZA. Apart from low activity retention in the liver and the bowel in the early phases, only blood pool activity was visible in healthy tissues in the scintigraphic images. In particular, no specific binding or persistent accumulation was apparent in the red bone marrow and the kidneys. The total body residence time was 2.2 ± 0.2 times higher for [^123^I]IMTO than for [^123^I]IMAZA; the residence time per liter of whole blood was 3.6 ± 1.5 times higher (Supplementary Fig. 2).

The plasma activity concentration was analyzed in patient #1 whose hematocrit was 35%. At 2 min after injection of [^123^I]IMAZA, 3.8% of the administered activity was found per liter of plasma, which corresponds to a distribution volume of 26 L. The activity concentration was 1.16 times higher in plasma than in the whole blood. These data are consistent with the assumption that [^123^I]IMAZA readily passes the membrane of red blood cells (plasma to whole blood activity concentration ratio expected from hematocrit, 1.13) and distributes to the rapidly exchangeable fraction of the total body water. After injection of [^123^I]IMTO, activity concentration in plasma was 1.16 times higher than in whole blood (Supplementary Fig. 2).

**Fig. 6:**
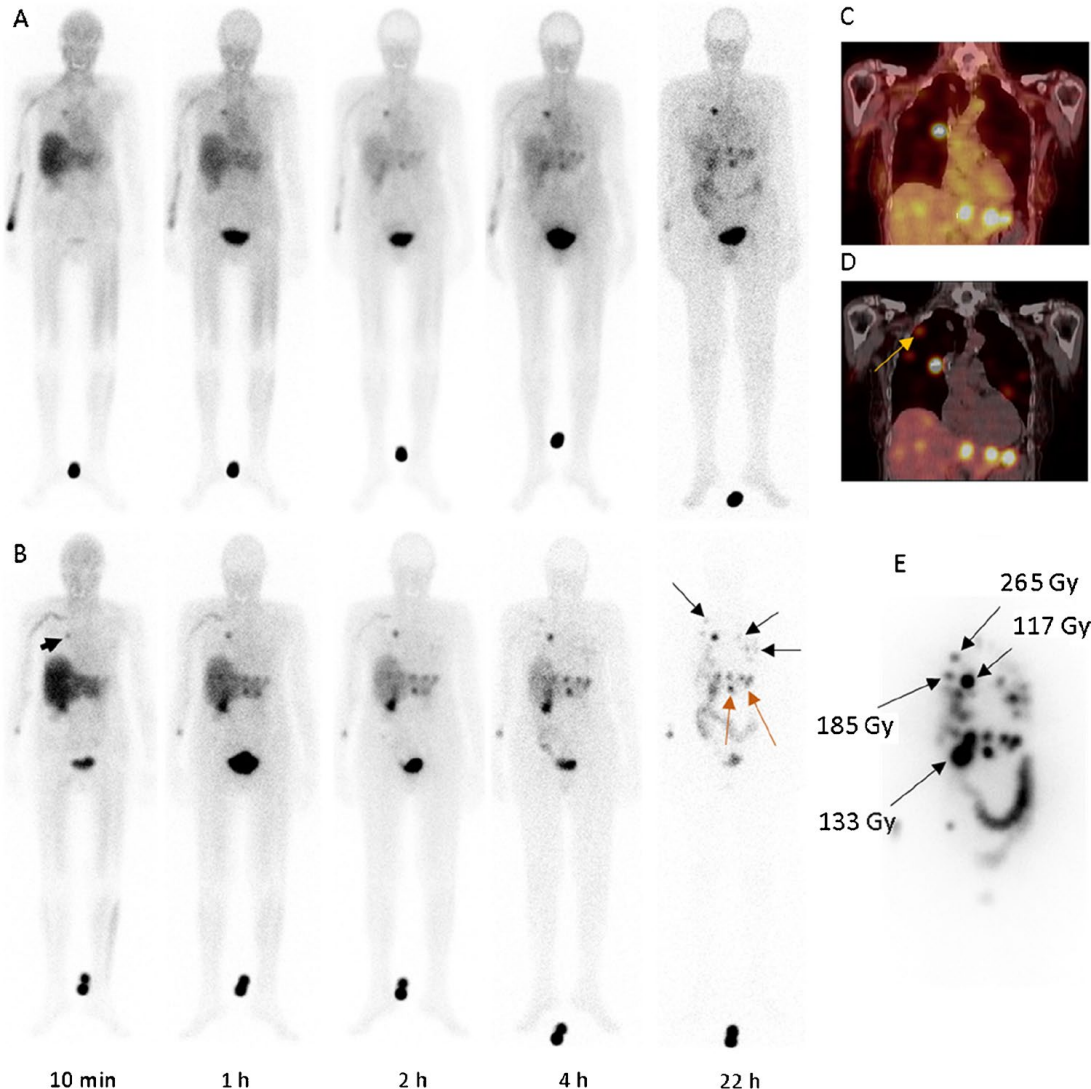
Scintigrams after i.v. injection of 176 MBq [^123^I]IMTO (**a** upper panel) and 117 MBq [^123^I]IMAZA (**b** lower panel), in a 73-year-old female (patient #1) with advanced, metastatic ACC. The largest lung metastasis in the right upper lobe (arrowhead) showed a high uptake already 10 min after tracer injection. A significantly lower background activity was found in [^123^I]IMAZA scans (**b**). The multiple lung metastases on both sides (black arrows) and hepatic metastases (red arrows) showed a higher uptake in the [^123^I]IMAZA late images compared with [^123^I]IMTO. **c** and **d** SPECT CT imaging with [^123^I]IMTO (**c**) and [^123^I]IMAZA (**d**) showing high and specific uptake of both tracers in the local recurrence and in liver metastases and pulmonal metastasis (yellow arrow). (**e**) Estimates for the mean absorbed tumor doses calculated from post-therapeutic imaging during the first and second treatment and lesion mass over the course of treatment

Uptake in large tumor lesions was visible already at 10 min after tracer injection with both [^123^I]IMTO and [^123^I]IMAZA. Specific tumor uptake was higher, and background activity was lower for [^123^I]IMAZA compared to [^123^I]IMTO (Fig. 6 and Supplementary Fig. 3). In 5 large lesions reliably measurable for both tracers, tumor uptake in the SPECT/CT imaging at 5.7 ± 0.1 h after the administration was a factor of 2.0 (range 1.5 to 2.8) higher for [^123^I]IMAZA, and the uptake to background ratio was higher by a factor of 4.6 (range 3.2 to 6.8). For both compounds, the effective half-life in tumor tissue was only slightly shorter than the physical half-life of I-123. The biological half-lives were too long to be derived reliably from the ^123^I data.

The residence time of the activity in the whole blood was shorter for [^131^I]IMAZA, and the kinetics recalculated for [^131^I]IMAZA corresponded to a mean blood absorbed dose of only 0.03 Gy per GBq compared to 0.11 Gy/GBq for [^131^I]IMTO. Areas under the curve of both tracers are presented in Supplementary Table 2.

### First treatment with [^131^I]IMAZA in a patient with metastatic ACC

In the pre-therapeutic kinetics assessment with [^131^I]IMAZA in patient #1, the residence time in the total body was 10.9 h and in the whole blood 0.17 h per liter of whole blood, indicating an absorbed dose to the blood of 0.032 Gy/GBq or 2 Gy after 63 GBq [^131^I]IMAZA. The corresponding values for [^131^I]IMTO, derived from diagnostics with [^123^I]IMTO, were much higher with 0.55 h residence time per liter of whole blood and a blood absorbed dose of 0.17 Gy/GBq.

Faint uptake in the liver and the renal pelvises was seen in the planar scintigraphic images up to 4 h. An absorbed dose of well below 0.2 Gy/GBq was estimated for both the liver and the kidney. In the later scans, the activity was only detected in the tumors except for a minor residual activity in the intestine, most likely due to additional biliary excretion.

The estimates for the mean absorbed tumor doses actually administered in the first therapy with 28.2 GBq [^131^I]IMAZA were 133 Gy for the recurrent primary and 117–265 Gy in 3 evaluable metastatic lesions (Fig. 6e). The absorbed dose to the blood was 1.1 Gy. In a second therapy course with 30.5 GBq [^123^I]IMAZA, the absorbed doses were 93 Gy to the primary and 145–210 Gy to the metastatic lesions. The absorbed dose to the blood was higher in the second treatment (1.7 Gy) due to a loss of renal clearance. The metastases known from the [^123^I]IMAZA scans show comparable high uptake in the dosimetric and post-therapeutic [^131^I]IMAZA scans; see Supplementary Fig. 4.

In our patient, we found a continuous decrease of metabolic activity and size of the tumor lesions over 8 months with a progression-free survival of 21 months and an overall survival of 30 months after initial endoradiotherapy.

## Discussion

Our article describes the successful development of a novel theranostic tool for adrenal tumors. In order to improve diagnostic imaging with [^123^I]IMTO and endoradiotherapy with [^131^I]IMTO, we started with the hypothesis that both higher avidity to the target enzymes and higher metabolic stability would result in an increased and longer lasting specific binding to target tissue and improved target to background ratios.

Starting from IMTO, a total of 50 new derivatives were synthesized and characterized. Of the most promising three new substances, we synthesized the corresponding trimethylstannyl precursors and used them to establish the radiosyntheses of the tracers. The chemistry and radiochemistry of the remaining new derivatives will be published elsewhere.

Cortisol and aldosterone synthesis were potently inhibited by the small aliphatic esters **3** and **4** and the small carboxylic acid amide IMAZA indicating high-affinity binding of these compounds to the respective enzymes. Considering the IC_50_ values for inhibition of cortisol and aldosterone secretion in the low nanomolar range up to only 0.3 nM, even higher affinity to the CYP11B enzymes compared to IMTO may be assumed for two agents.

In vitro analysis of metabolic inactivation after preincubation with human liver microsomes revealed only three compounds with comparable (**3**, **4**) or higher (IMAZA) stability than IMTO. It is noteworthy that these three compounds have comparable (**3**, 4) or lower (IMAZA) lipophilicity estimated from HPLC retention times. This fits well to the observation that the binding sites of metabolizing enzymes are generally lipophilic in nature [25]. Apart from the lower lipophilicity, the higher stability can be explained by the carboxylic amide as functional group and by the cyclic structure of IMAZA [26, 27].

The target enzymes CYP11B1 and CYP11B2 are localized at the mitochondrial inner membrane, so penetrance of the cell membranes is a prerequisite for target binding. Cellular subfractionation further revealed strong uptake in the mitochondrial fraction. Both cell uptake and accumulation in the mitochondrial subfraction could be blocked by co-incubation with etomidate indicating specific CYP11B binding (Fig. 3b).

In biodistribution experiments, all radiolabeled compounds showed highly specific binding to adrenal tissue (Fig. 4B). [^125^I]**3**, [^125^I]**4**, and [^125^I]IMAZA showed higher tracer uptake with better target to background ratios than observed for [^125^I]IMTO. For [^125^I]IMAZA, uptake at the latest time point was 10 times higher compared to [^125^I]IMTO in mice.

Subsequent binding studies of [^125^I]IMAZA to human adrenocortical tumors also revealed strong and specific binding to adrenocortical tissue sections (Fig. 5), corroborating the results from our mouse experiments. These observations were further supported by our first clinical application of both [^123^I]IMTO and [^123^I]IMAZA scintigraphy in three patients with metastatic adrenocortical carcinoma. In these patients, tumor uptake was about two times higher at 6 h after administration of [^123^I]IMAZA, and the target tissue to background ratio was higher by a factor of 4.6 compared to [^123^I]IMTO.

In direct comparison, [^123^I]IMAZA provided much higher tumor to background ratios than [^123^I]IMTO, and a rapid clearance from the non-target tissue was observed (Fig. 6). Unbound [^123^I]IMAZA was completely eliminated from the non-target tissue after 24 h. These specific properties might translate into a better diagnostic sensitivity. Accordingly, in our patient, several lesions were visualized by [^123^I]IMAZA but could not be detected by [^123^I]IMTO imaging (Fig. 6a–d).

The rapid clearance further results in a lower dose to both the whole body and the red bone marrow which is the critical dose-limiting organ. The low residence time of the activity in the whole blood for [^131^I]IMAZA enables an extraordinarily high treatment activity for [^131^I]IMAZA of 60 GBq. The tumor doses achieved by treatment of patient #1 with [^131^I]IMAZA were up to 265 Gy and thus several times higher than the tumor doses observed in patients treated with [^131^I]IMTO, being below 50 Gy [24]. They were furthermore reached after administration of 28.2 GBq, which is only half of the maximum possible activity. The long-term disease stabilization of 21 months and survival of 30 months after a rapid increase of the tumor masses before first treatment with [^131^I]IMAZA demonstrate the treatment efficacy of our new radiopharmaceutical.

## Conclusion

In summary, [^123/131^I]IMAZA holds great potential to further improve the diagnostic evaluation of adrenal tumors and of metastatic ACC but also enables endoradiotherapy of advanced ACC.

## Supplementary Information

Below is the link to the electronic supplementary material.

Supplementary file1 (DOCX 3312 KB)

## Data Availability

Not applicable.
