# Supplementary material for: Novel CYP11B-ligand [123/131I]IMAZA as promising theranostic tool for adrenocortical tumors: comprehensive preclinical characterization and first clinical experience
Source: Eur J Nucl Med Mol Imaging. 2021 Jul 3;49(1):301–10. doi: 10.1007/s00259-021-05477-y (PMC8712301; doi:10.1007/s00259-021-05477-y)
Supplement: Supplementary file 1 — Supplementary file1 (DOCX 3312 KB) [file 259_2021_5477_MOESM1_ESM.docx]

**Chemistry**

**Reagents and materials**

Solvents and reagents were purchased from commercial sources in the highest purity available and used without further purification. ^1^H-NMR spectra were recorded on a Bruker AM 400 spectrometer in CDCl_3_ using the residual solvent peak as internal reference [δ(CHCl_3_) = 7.26; δ(DMSO) = 2.54]. Chemical shifts, δ, are given in parts per million. Melting points are uncorrected.

**Preparation of IMTO and novel Derivatives**

For the preparation of the new derivatives, large amounts of IMTO were required as an intermediate. Direct electrophilic iodination of commercially available metomidate results in a difficult to separate mixture of isomers [Hammerschmidt F. et al. Monatsh. Chem. 2005, **136**, 229–239.]. Therefore, Hammerschmidt et al. developed a chemoenzymatic synthesis of IMTO with a lipase catalysed enantioselective hydrolysis as a key step. In order to avoid the resolution of racemic compounds, we adapted a stereoselective multistep synthesis of metomidate [Kazmirowski H.G. et al. Patent DD 205683 A1 19840104.]. For this purpose, we selectively iodinated inexpensive R-(+)-1-phenylethylamine in the 4-position, as was already described for the (S)-enantiomer [McKittrick B.A. et al. Patent WO2002051809 p.11.]. The resulting R-(+)-1-(4-Iodophenyl)-ethylamine was transferred into multigram amounts of IMTO via a five step sequence including alkylation, formylation, ring-closure, and oxidative desulfurization (Scheme 1). After saponification of IMTO to the corresponding acid and further treatment with thionyl chloride or oxalyl chloride to the acid chloride the conversion to carboxylic acid esters and carboxylic amides failed in our hands. Therefore, the acid was converted with the help of the coupling agents 1-ethyl-3-(3'-dimethyl-aminopropyl)carbodiimide/1-hydroxybenzotriazole (EDC/HOBt) into the esters [Chan L.C. et al. J. Org. Chem. 2007, **72**, 8863-8869.] and trimethylphosphite/iodine was used for the preparation of the carboxylic amides [Morcillo S.P. et al. J. Org. Chem. 2011, **76**, 2277-2281]. Synthesis of the corresponding trimethylstannyl precursors was performed by the Pd-catalyzed reaction of the iodinated compounds with hexamethylditin.

Scheme 1. Synthesis of IMTO and novel derivatives

Reagents: (a) TFAA, I_2_, TFAI; (b) NaOH; (c) methyl chloroacetate; (d) formic acid; (e) methyl formiate, sodium methylate; (f) KSCN; (g) HNO_3_; (h) NaOH; (i) azetidine, P(OMe)_3_, I_2_, NEt_3_; (j) alcohol, EDC, HOBt; (k) Sn_2_(CH_3_)_6_, NEt_3_, Pd(PPh_3_)_3_.

**(R)-1-[1-(4-Iodophenyl)ethyl]-1H-imidazole-5-carboxylic acid methyl ester (Iodometomidate, IMTO) 1**

IMTO was prepared as described above and already published in detail [Allolio B. et al. Patent WO 2014048568].

White solid, mp 72-4°C, yield: 13.7 g (38.5 mmol, 40.9%)

TLC (silica gel): R_f_ (heptane/ethyl acetate 80/20): 0.10

MS, *m/e* 356.02 (M^+^)

^1^H-NMR (CDCl_3_): δ = 7.80 (s, 1H), 7.78 (s, 1H), 7.67 (d, 2H), 6.92 (d, 2H), 6.30 (q, 1H), 3.81 (s, 3H), 1.87 (d, 3H)

**(R)-1-[1-(4-Iodophenyl)ethyl]-1H-imidazole-5-carboxylic acid 2**

A solution of 4 g (11 mmol) IMTO in 75 mL 10% NaOH was refluxed for 5 h. After cooling to rt a pH-value of 2-3 was adjusted by addition of conc. HCl. The precipitating product was filtered off and washed with water. The crude product was suspended in 100 ml toluene and dried by azeotropic distillation using a dean-stark trap. After cooling the solvent was stripped and the product was dried in a desiccator.

White solid, mp > 250°C (dec.), yield: 3.32 g (9.70 mmol, 88.2%)

TLC (silica gel): R_f_ (heptane/ethyl acetate 80/20): 0.00

^1^H-NMR (d_6_-DMSO): δ = 8.27 (s, 1H), 7.73 (d, 2H) 7.65 (s, 1H), 6.95 (d, 2H), 6.30 (q, 1H), 1.80 (d, 3H)

**(R)-1-[1-(4-Iodophenyl)ethyl]-1H-imidazole-5-carboxylic acid azetidinylamide (IMAZA) 5**

A solution of 534 µL (558 mg, 4.5 mmol) trimethylphosphite in 24 mL methylene chloride was cooled in an ice bath and was charged with 1.14 g (4.5 mmol) iodine. After complete dissolution of the iodine, 1.55 g (4.5 mmol) **2** and 1.04 mL (759 mg, 7.5 mmol) triethylamine were added. 10 min later, 507 µL (429 mg, 7.5 mmol) azetidine was added and the solution stirred for 3 h at room temperature. The solution was diluted with 100 mL chloroform, washed once with 100 mL of a saturated sodium thiosulfate-solution and three times with each 50 mL water and dried over sodium sulfate. After stripping the solvent, the crude product was purified by column chromatography (CH_2_Cl_2_/CH_3_OH 95/5).

Yellow waxy solid, yield: 770 mg (2.02 mmol, 44.9%)

TLC (silica gel): R_f_ (CH_2_Cl_2_/CH_3_OH 95/5) = 0.30

MS *m/e* 381.9 (M^+^)

^1^H-NMR (CDCl_3_): δ = 7.73 (s, 1H), 7.63 (d, 2H), 7.31 (s, 1H), 6.90 (d, 2H), 6.43 (q, 1H), 4.00-4.40 (m, 4H), 2.20-2.40 (m, 2H), 1.82 (d, 3H)

**(R)-1-[1-(4-Iodophenyl)ethyl]-1H-imidazole-5-carboxylic acid 3-tetrahydrofurylester 3**

To a solution of 808 µL (881 mg, 10 mmol) 3-hydroxytetrahydrofuran in 1 mL dry methylene chloride was added 350 mg (1.03 mmol) **2** and stirred for 5 min at rt. To the resulting reaction mixture HOBt (1-hydroxybenzotriazole) (94 mg, 0.7 mmol) and NMM (N-methyl morpholine) (209 mg, 2.06 mmol) were added. After 30 min., EDC (1-(3-dimethylaminopropyl)-3-ethylcarbodiimide) (320 mg, 4.12 mmol) was added to reaction mixture and stirred for 2 d at rt. The mixture was diluted with water; organic layer was extracted and concentrated in vacuum. Crude product was chromatographed on a silica gel column using MeOH/DCM (3:97) as eluent to provide the product.

Yellow oil, yield: 237 mg (0.57 mmol, 55.3%)

TLC (silica gel): R_f_ (heptane/ethyl acetate 80/20): 0.05

MS *m/e* 413.16389 (M + H)

^1^H-NMR (CDCl_3_): δ = 7.69 (m, 2H), 7.59 (d, 2H), 6.82 (m, 2H), 6.17 (q, 1H), 5.33 (m, 1H), 3.83 (m, 4H), 2.14 (m, 1H), 2.00 (m, 1H), 1.76 (d, 3H)

**(R)-1-[1-(4-Iodophenyl)ethyl]-1H-imidazole-5-carboxylic acid 2,2-difluoroethylester 4**

The synthesis of 33 was performed using the same protocol used in the synthesis of **3**.

Pale yellow oil, yield: 241 mg (0.59 mmol, 57.6%)

TLC (silica gel): R_f_ (heptane/ethyl acetate 80/20): 0.15

MS *m/e* 407.00796 (M + H)

^1^H-NMR (CDCl_3_): δ = 7.85 (s, 1H), 7.79 (s, 1H), 7.65 (d, 2H), 6.89 (d, 2H), 6.20 (q, 1H), 5.85-6.11 (m, 1H), 4.38 (m, 2H), 1.86 (d, 3H)

**General method for stannylation**

To a solution of 0.76 mmol of the iodinated arene in 12 mL toluene 728 mg (2.26 mmol) hexamethylditin, 67 mg (0.055 mmol) Tetrakis-(triphenylphosphine)-palladium (0) and 1.80 mL (1.30 g, 13.0 mmol) triethylamine were added. The solution was refluxed overnight, the solvent stripped and the crude product purified by column chromatography (heptane/EtOAc 50/50 – 0/100).

**(R)-1-[1-(4-Trimethylstannylphenyl)ethyl]-1H-imidazole-5-carboxylic acid methyl ester**

White solid, m.p. 77-79°C, yield: 188 mg (0.48 mmol, 63.0%)

TLC (silica gel): R_f_ (heptane/ethyl acetate 80/20): 0.15

MS *m/e* 394.09 (M^+^)

^119^Sn-NMR (CDCl_3_): δ = -26.2

^1^H-NMR (CDCl_3_): δ = 7.75 (s, 1H), 7.72 (s,1H), 7.43 (d, 2H), 7.13 (d, 2H), 6.30 (q, 1H), 3.77 (s, 3H), 1.82 (d, 3H), 0.25 (s, 9H)

**(R)-1-[1-(4-Trimethylstannylphenyl)ethyl]-1H-imidazole-5-carboxylic acid azetidinylamid**

Yellow waxy solid, yield: 125 mg (0.30 mmol, 59.8%)

TLC (silica gel): R_f_ (CH_2_Cl_2_/CH_3_OH 95/5) = 0.25

MS *m/e* (M + H) 420.11180

^1^H-NMR (CDCl_3_): δ = 7.72 (s, 1H), 7.43 (d, 2H), 7.25 (s, 1H), 7.19 (d, 2H), 6.43 (q, 1H), 4.00-4.35 (m, 4H), 2.20-2.35 (m, 2H), 1.82 (d, 3H), 0.28 (s, 9 H)

**(R)-1-[1-(4-Trimethylstannylphenyl)ethyl]-1H-imidazole-5-carboxylic acid 3-tetrahydrofuryl ester**

Yellow oil, yield: 47 mg (0.11 mmol, 14.5%)

TLC (silica gel): R_f_ (heptane/ethyl acetate 80/20): 0.15

MS *m/e* 451.10304 (M + H)

^1^H-NMR (CDCl3): δ = 7.70 (s,1H), 7.68 (s, 1H), 7.59 (d, 2H), 6.82 (d, 2H), 6.17 (q, 1H), 5.33 (m, 1H), 3.83 (m, 4H), 2.14 (m, 1H), 2.00 (m, 1H), 1.76 (d, 3H), 0.27 (s, 9H)

**(R)-1-[1-(4-Trimethylstannylphenyl)ethyl]-1H-imidazole-5-carboxylic acid 2,2-difluoroethyl ester**

Yellow oil, yield: 92 mg (0.21 mmol, 27.6%)

TLC (silica gel): R_f_ (heptane/ethyl acetate 80/20): 0.20

MS *m/e* 467.05775 (M + Na)

^1^H-NMR (CDCl_3_): δ = 7.86 (s, 1H), 7.81 (s, 1H), 7.47 (d, 2H), 7.14 (d, 2H), 6.27 (q, 1H), 5.85-6.13 (m, 1H), 4.39 (m, 2H), 1.85 (d, 3H), 0.29 (s, 9H)

**Radiochemistry**

Radio-HPLC was performed on a system consisting of a Shimadzu pump (LC-10AT) and a Shimadzu UV/vis detector (SPD-10A) with a wavelength of 254 nm. Sample injection was accomplished by a Rheodyne-Injector-block (7725i). For measurement of radioactivity the outlet of the UV detector was connected to a NaI(Tl) scintillation detector (Berthold LB 1200) and the recorded data was processed by a software system (Shimadzu Class-VP). Puriﬁcation of the tracers was performed using a C18 phase (Nucleosil 100-7 250×4.6mm, CS Chromatographie Service, Langerwehe, Germany), eluent: CH_3_OH/H_2_O/triethylamine 70/30/0.1 v/v/v and a flow of 1.0 mL/min.

**Radiosynthesis and quality control of [^123^I]IMAZA for scintigraphy of patients**

Labeling was performed in the delivered vial containing [^123^I]NaI in about 10 µL 0.02 N NaOH (CYI.9; GE Healthcare, Braunschweig, Germany). To this vial a solution of 40 µg of the stannylated precursor in 30 µL ethanol were pipetted followed by 6 µl 1 N HCl and 10 µl oxidizing agent (15 mg Chloramine-T trihydrate in 10 ml water). The reaction was allowed to proceed at room temperature for 3 min and quenched by the addition of 10 µl of a reduction agent (40 mg Na_2_S_2_O_5_ in 10 ml water). After labelling, the solution was directly injected into the HPLC-system. Radio-HPLC was performed on a system consisting of a Shimadzu pump (LC-10AT) and a Shimadzu UV/vis detector (SPD-10A) with a wavelength of 230 nm. Sample injection was accomplished by a Rheodyne-Injector-block (7725i). For measurement of radioactivity the outlet of the UV detector was connected to a NaI(Tl) scintillation detector (Berthold LB 1200) and the recorded data was processed by a software system (Shimadzu Class-VP). Purification of [^123^I]IMAZA was performed using a C18 phase (Kromasil 100-5 250 x 4.6 mm, CS Chromatographie Service, Langerwehe, Germany), eluent: EtOH/H_2_O/H_3_PO_4_ (25%) 150/350/0.2 (v/v/v), pH-value: <7, flow: 1.0 mL/min. Retention time of [^123^I]IMAZA: ~ 12 min.

The [^123^I]IMAZA containing HPLC fraction (radiochemical yield > 90%) is transferred into a sterile bench, diluted with 1 ml PBS concentrate to adjust the pH to 7.4, and further diluted with saline to the double volume of the initial HPLC fraction. The ethanol content is therefore adjusted to about 15%. For i.v.-injection, the solution was passed through a sterile 0.22 µm Millipore filter into a sterile vial.

Quality control was performed using another HPLC-system consisting of the same hardware and equipped with a Nucleosil 100-10-column, 250 x 4.6 mm (CS Chromatographie Service, Langerwehe, Germany), eluent: CH_3_OH/H_2_O/diethylamine 60/40/0.2 (v/v/v), flow: 1.0 mL/min. Retention time of [^123^I]IMAZA: ~ 6 min. The sample was coinjected with 10 µg of IMAZA; the difference in the retention times of 0.4 min is caused by non-simultaneous acquisition of the data in the radioactivity and UV-channel. Molar activity of [^123^I]IMAZA was determined to 306,200 ± 60,200 MBq/µmol.

**Radiosynthesis of [^131^I]IMAZA for endoradiotherapy of patients**

The radiosynthesis of [^131^I]IMAZA was performed by a GRP-module (Scintomics GmbH, Fürstenfeldbruck, Germany) inside a well-ventilated lead cell. Briefly, to a lead-shielded vial containing up to 33.3 GBq [^131^I]iodide in 1 ml 0.01 N NaOH (IBSSO; GE Healthcare, Braunschweig, Germany), a solution of 5 mg of the corresponding trimethylstannyl precursor in 1 ml ethanol, 120 µl 2 N HCl, and a solution of 2.25 mg Chloramine-T trihydrate in 150 µl water were consecutively added with a syringe. After a reaction time of 3 min at room temperature, 135 µl 2 N NaOH and a solution of 4.5 mg Na_2_S_2_O_5_ in 150 µl water were added, and the solution was automatically injected into a HPLC system equipped with a semipreparative RP-18 column (Nucleosil 100–5C18, 250 x 8 mm; CS Chromatographie Service, Langerwehe, Germany). HPLC separation was performed using ethanol/PBS 40/60 as eluent with a flow of 2.0 ml/min. Retention time of [^131^I]IMAZA: ~ 50 min.

The [^131^I]IMAZA containing fraction (radiochemical yield 85-90%) was collected, passed through a sterile filter (0.22 µm), and - directly after quality control - used for administration. Quality control was performed in the same HPLC-system and conditions used for [^123^I]IMAZA. Molar activity of [^131^I]IMAZA was determined to 64,000 ± 14,000 MBq/µmol.

**Radiosynthesis and quality control of [^123^I]IMTO for scintigraphy of patients**

Labeling of [^123^I]IMTO was performed using the same protocol given for the radiosynthesis of [^123^I]IMAZA, but using only 30 µg of the stannylated precursor. Purification of [^123^I]IMTO was performed using a C18 phase (Kromasil 100-5 250 x 4.6 mm, CS Chromatographie Service, Langerwehe, Germany), eluent: EtOH/H_2_O/ammonia (25%) 50/50/0.1 v/v/v, pH-value: <7, flow: 1.0 mL/min. Retention time of [^123^I]IMTO: ~ 19 min..

The [^123^I]IMTO containing HPLC fraction (radiochemical yield > 90%) was evaporated to dryness at room temperature under reduced pressure. Heating during this step should be strictly avoided in order to suppress deiodination and to maintain high radiochemical purity. For i.v.-injection, the residue was redissolved in a suitable volume of PBS containing 20% ethanol and passed through a sterile 0.22 µm filter into a sterile vial. Quality control as described for [^123^I]IMAZA eluent: CH_3_OH/H_2_O/diethylamine 70/30/0.2 (v/v/v), flow: 1.0 mL/min. Retention time of [^123^I]IMTO: ~ 6 min. The sample was coinjected with 10 µg of IMTO; the difference in the retention times of 0.4 min is caused by non-simultaneous acquisition of the data in the radioactivity and UV-channel. Molar activity of [^123^I]IMTO was determined to 175,400 ± 48,200 MBq/µmol.

**Radiosynthesis and quality control of [^125^I]3**

Labeling of [^125^I]**3** was performed using the same protocol given for the radiosynthesis of [^123^I]IMTO, using only 30 µg of the stannylated precursor and n.c.a. [^125^I]NaI in 0.01 N NaOH (Biotrend, Cologne, Germany). Purification of [^125^I]**3** was performed using a C18 phase (Nucleosil 100-7 250×4.6mm, CS Chromatographie Service, Langerwehe, Germany), eluent: CH_3_OH/H_2_O/triethylamine 70/30/0.1 v/v/v and a flow of 1.0 mL/min.

**
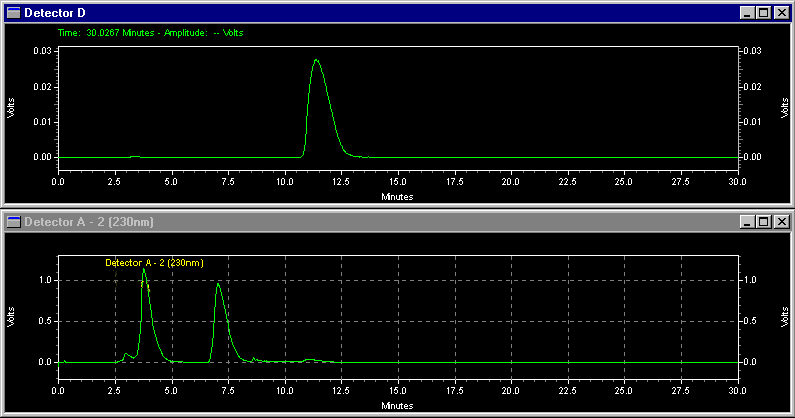
**

The [^125^I]**3** containing HPLC fraction (radiochemical yield > 90%) was evaporated to dryness at room temperature under reduced pressure. The residue was redissolved in a suitable volume of PBS containing 20% ethanol. Quality control was performed using a C18 phase (Nucleosil 100-10 250×4.6mm: CH_3_OH/H_2_O/diethylamine 70/30/0.2 (v/v/v), flow: 1.0 mL/min. The sample was coinjected with 10 µg of **3**.

**Radiosynthesis and quality control of [^125^I]4**

Labeling and purification of [^125^I]**4** was performed using the same protocol given for the radiosynthesis of [^125^I]**3**.

**
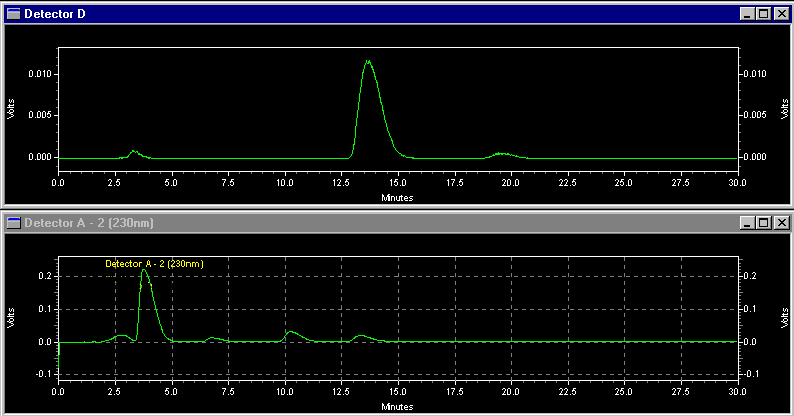
**

The [^125^I]**4** containing HPLC fraction (radiochemical yield > 90%) was evaporated to dryness at room temperature under reduced pressure. The residue was redissolved in a suitable volume of PBS containing 20% ethanol. Quality control was performed using a C18 phase (Nucleosil 100-10 250×4.6mm: CH_3_OH/H_2_O/diethylamine 75/25/0.2 (v/v/v), flow: 1.0 mL/min. The sample was coinjected with 10 µg of **4**.

**Analysis of metabolic inactivation by HPLC**

Radio-HPLC was performed on a Shimadzu system consisting of a LC-10AT pump, a CBM-10A controller and a FRC-10A fraction collector and a Rheodyne-Injector-block (7725i). Analysis was performed using a C18 phase (Nucleosil 100-10 C18 250 x 4.6 mm, CS Chromatographie Service, Langerwehe, Germany), eluent: CH_3_OH/H_2_O/NH_3_ 70/30/0.1 (v/v/v), pH-value: >7, flow: 1.0 mL/min for 20 min and the HPLC-fractions (1 mL each) were automatically collected in plastic test tubes. The collected HPLC fractions were measured in a gamma counter and the radioactivity of the tracer fraction was set in relation to the total radioactivity. Subsequently, the logarithm of the fraction of unmetabolized activity was plotted versus time, and the half-life time was obtained from the slope (see Supplementary Figure 1 for [^125^I]IMAZA).

**Supplementary Figure 1** Determination of the metabolic stability of [^125^I]IMAZA

**Supplementary Table 1** Uptake of four ^125^I-labeled tracers in the adrenals of male CD-1 mice at four different time points.

|  | **[^125^I]IMTO** | **[^125^I]IMAZA** | **[^125^I]4** | **[^125^I]3** |
| --- | --- | --- | --- | --- |
| **15 min** | 491.2 ± 170.3 | 710.9 ± 210.5 | 590.6 ± 250.4 | 360.3 ± 108.7 |
| **30 min** | 321.3 ± 170.6 | 720.4 ± 169.7 | 291.2 ± 97.5 | 240.4 ± 135.9 |
| **120 min** | 80.5 ± 43.7 | 395.7 ± 92.7 | 49.7 ± 13.1 | 61.5 ± 30.1 |
| **240 min** | 27.4 ± 25.9 | 275.4 ± 124.1 | 32.7 ± 17.1 | 61.4 ± 48.5 |

**
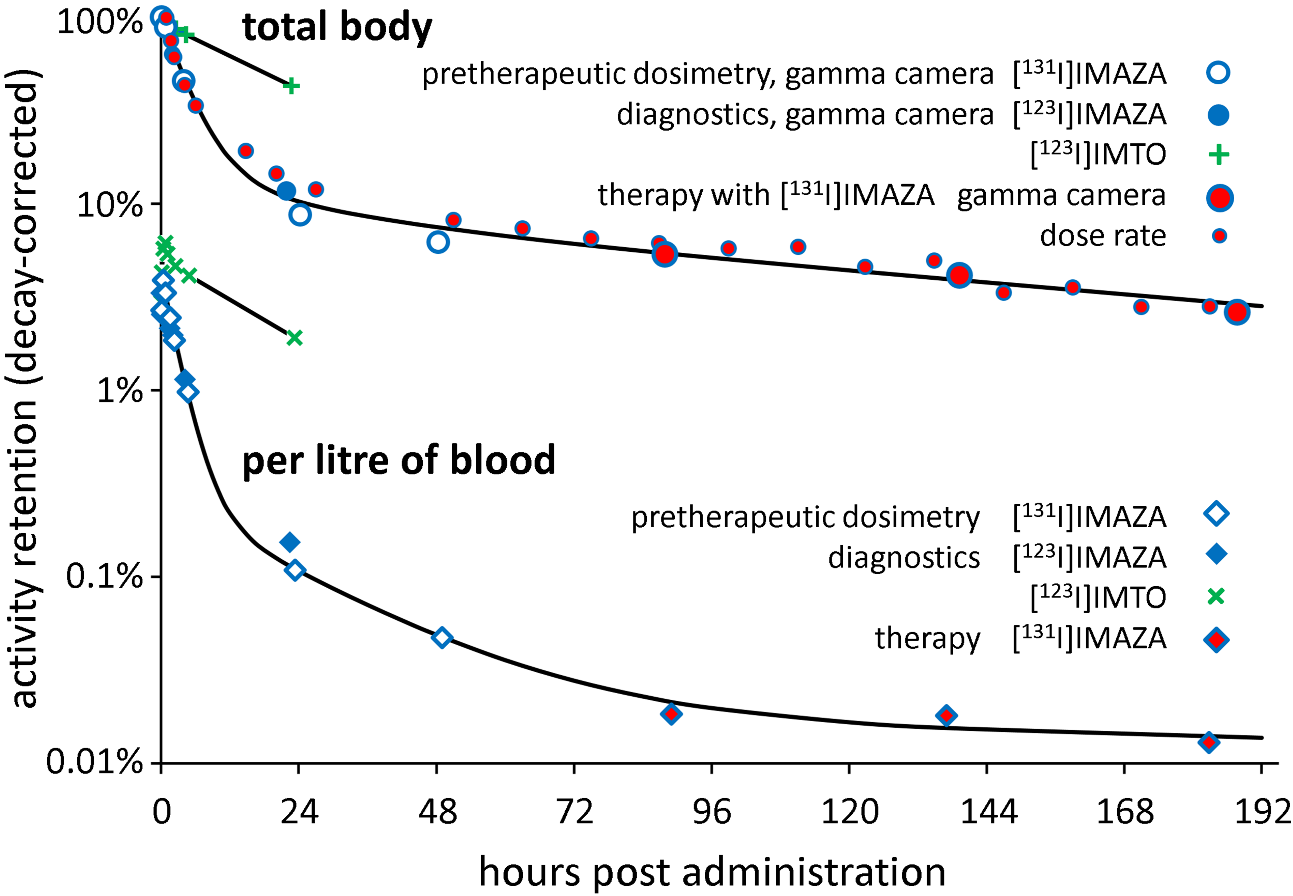
**

**
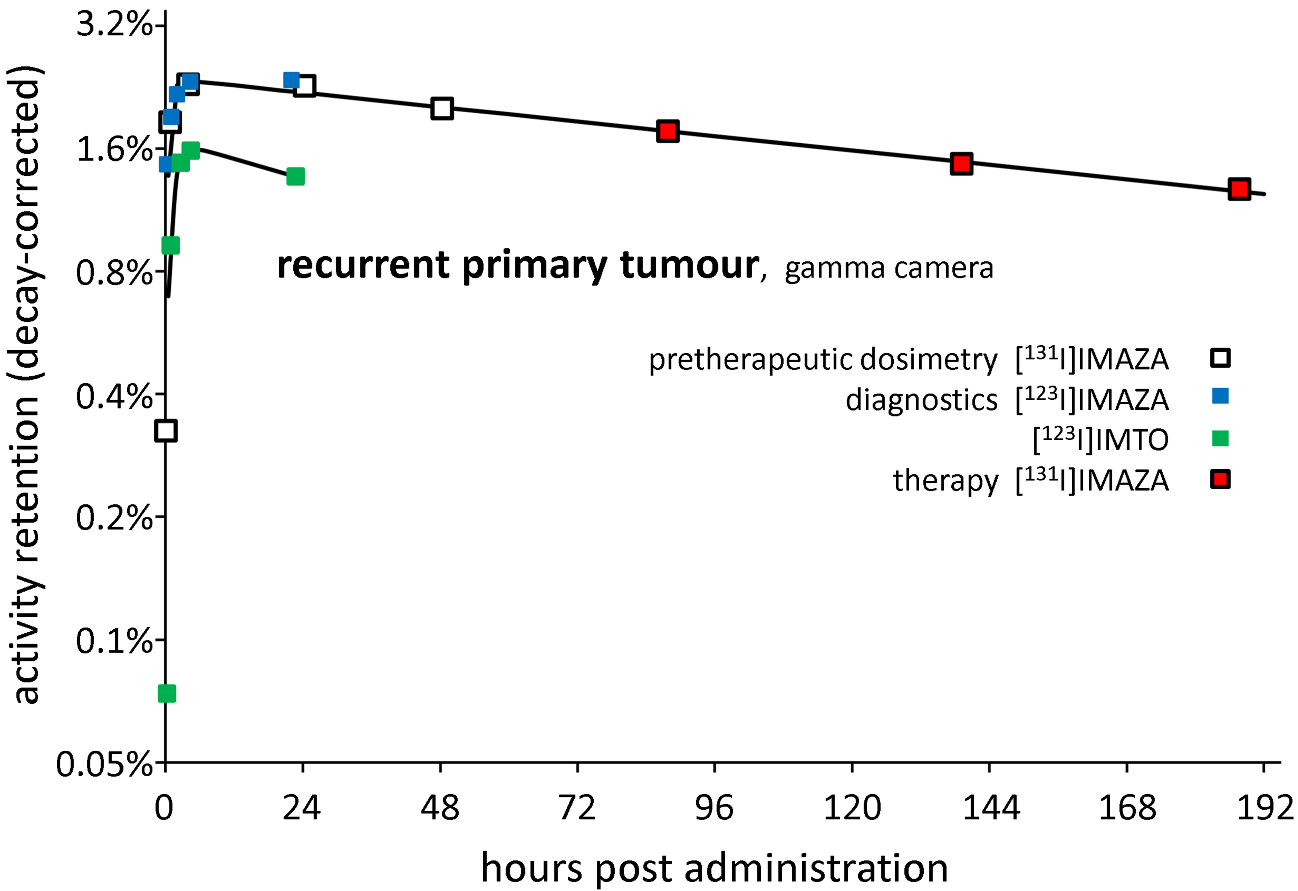
**

**Supplementary Fig. 2** Biokinetics in Patient #1. The upper diagram shows the activity retention corrected for physical decay in total body and per litre of whole blood, the lower diagram the retention in the recurrent primary tumor (lesion #1,#1 in Supplementary Table 2). Symbols show measured data after diagnostics with [^123^I]IMTO (green) or [^123^I]IMAZA (blue), after pre-therapeutic dosimetry with 74 MBq [^131^I]IMAZA (white), and after therapy with 28.2 GBq [^131^I]IMAZA (red). Lines show fit functions.


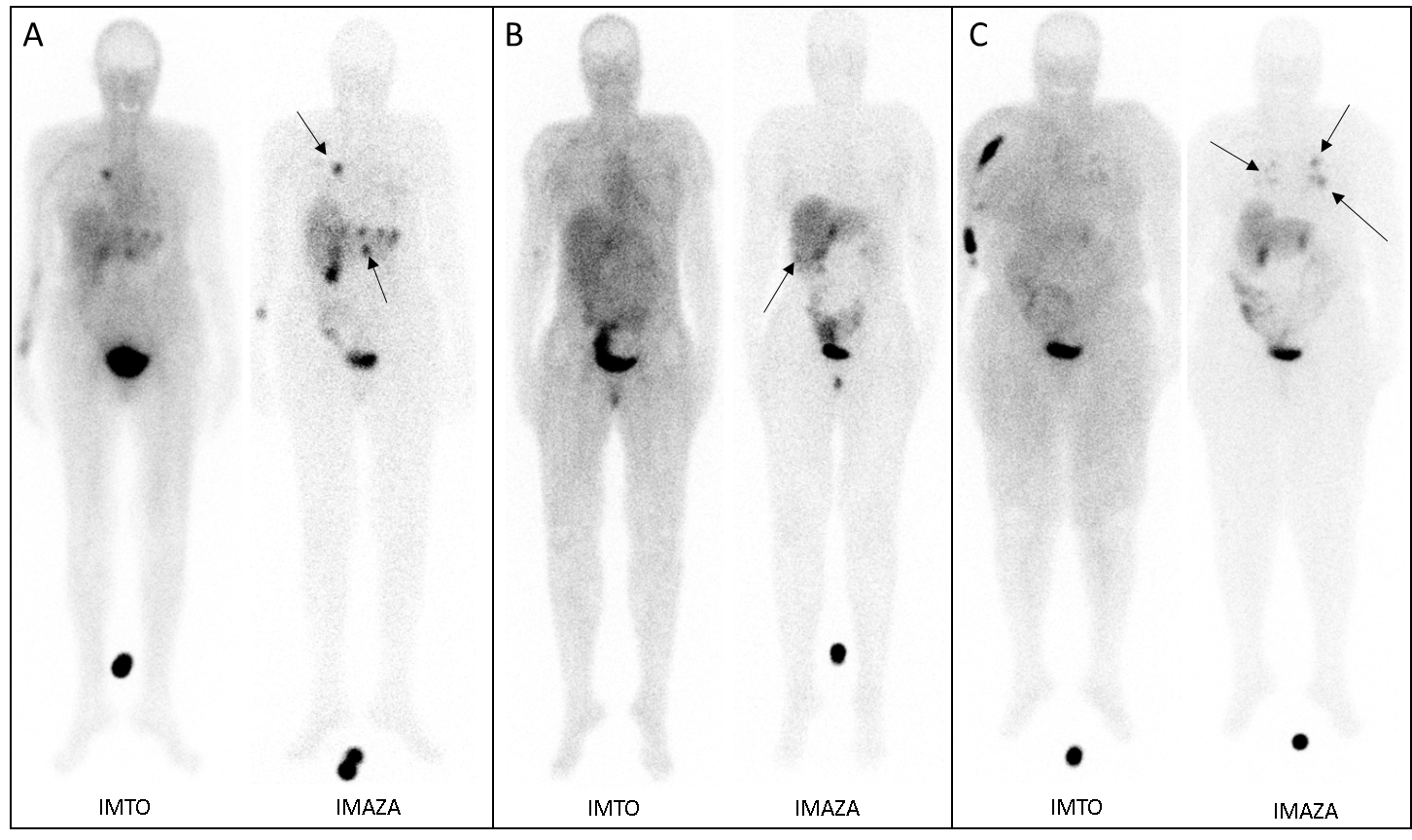


**Supplementary Figure 3** Comparisons of scintigrams of three patients (**a**, **b**, **c**) undergoing [^123^I]IMTO and [^123^I]IMAZA imaging after 4 h. A significantly lower background activity was found in the [^123^I]IMAZA scans. The arrows highlight exemplary some metastases which showed a higher uptake for [^123^I]IMAZA.

Patient #1 (**a**) received 176 MBq [^123^I]IMTO and 117 MBq [^123^I]IMAZA.

Patient #2 (**b**) received 171 MBq [^123^I]IMTO and 167 MBq [^123^I]IMAZA.

Patient #3 (**c**) received 145 MBq [^123^I]IMTO and 183 MBq [^123^I]IMAZA.

**Supplementary Table2** Characteristics of [^123^I]IMAZA and [^123^I]IMTO in five tumor lesions.

|  | Patient, lesion | #1, #1 | #1, #2 | #2, #1 | #3, #1 | #3, #2 | Median |
| --- | --- | --- | --- | --- | --- | --- | --- |
| [^123^I]IMAZA | time of SPECT/CT (h) | 5.6 | 5.6 | 5.9 | 5.6 | 5.6 | 5.6 |
|  | uptake (%/g) | 0.021 | 0.017 | 0.016 | 0.027 | 0.041 | 0.021 |
|  | effective half-live (h) | 13.2 | 13.2 | 11.1 | 6.6 | 11.3 | 11.3 |
|  | AUC (h/g) | 0.0054 | 0.0041 | 0.0036 | 0.0044 | 0.0093 | 0.0044 |
| [^123^I]IMTO | time of SPECT/CT (h) | 5.6 | 5.6 | 5.8 | 5.5 | 5.5 | 5.6 |
|  | uptake (%/g) | 0.015 | 0.011 | 0.006 | 0.010 | 0.027 | 0.011 |
|  | effective half-live (h) | 11.2 | 11.0 | 12.4 | 8.0 | 10.1 | 11.0 |
|  | AUC (h/g) | 0.0031 | 0.0024 | 0.0016 | 0.0017 | 0.0056 | 0.0024 |
| Ratio | uptake (%/g) | 1.45 | 1.48 | 2.53 | 2.75 | 1.52 | 1.52 |
|  | effective half-live (h) | 1.18 | 1.20 | 0.90 | 0.83 | 1.12 | 1.12 |
|  | AUC (h/g) | 1.71 | 1.75 | 2.28 | 2.52 | 1.67 | 1.75 |

uptake: activity concentration measured in SPECT/CT normalised to the administered activity

AUC: time integral of the uptake function (area under the curve)


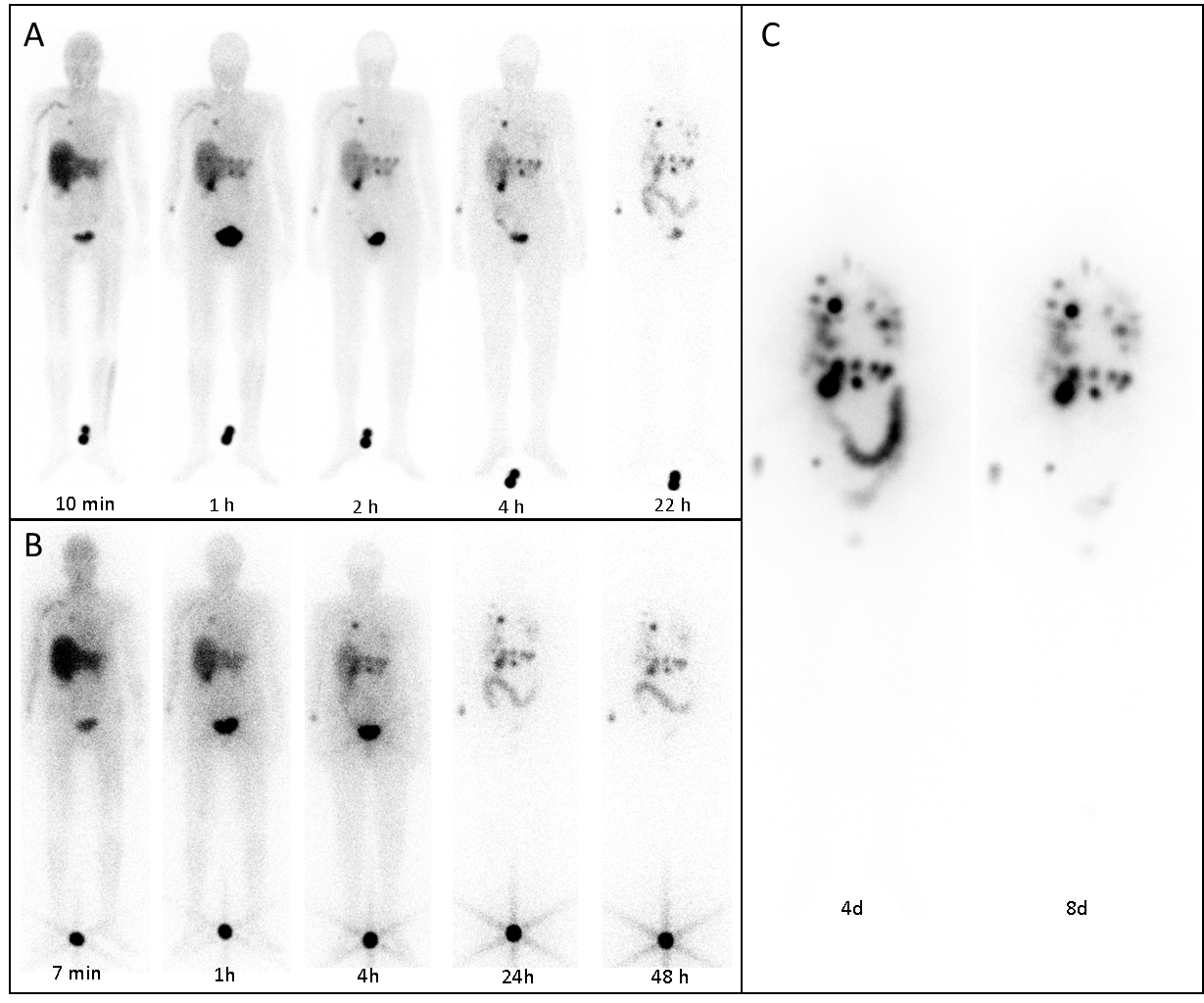


**Supplementary Figure 4** Scintigrams after i.v. injection of 117 MBq [^123^I]IMAZA (**a** upper panel) and 73.9 MBq [^131^I]IMAZA (**b** lower panel), in a 73 year old female (patient #1) with advanced, metastatic ACC. The metastases known from the [^123^I]IMAZA scans show comparable high uptake in the dosimetric [^131^I]IMAZA scans.

Posttherapeutic scans (**c**) after application of 28.3 GBq [^131^I]IMAZA show concordant uptake in the preknown metastases with a very long half-life, indicated by a high retention after four and eight days.
